# Deep Cross-User Models Reduce the Training Burden in Myoelectric Control

**DOI:** 10.3389/fnins.2021.657958

**Published:** 2021-05-24

**Authors:** Evan Campbell, Angkoon Phinyomark, Erik Scheme

**Affiliations:** Department of Electrical and Computer Engineering, Institute of Biomedical Engineering, University of New Brunswick, Fredericton, NB, Canada

**Keywords:** EMG, gesture recognition, deep learning, domain adaptation, cross-user, training burden

## Abstract

The effort, focus, and time to collect data and train EMG pattern recognition systems is one of the largest barriers to their widespread adoption in commercial applications. In addition to multiple repetitions of motions, including exemplars of confounding factors during the training protocol has been shown to be critical for robust machine learning models. This added training burden is prohibitive for most regular use cases, so cross-user models have been proposed that could leverage inter-repetition variability supplied by other users. Existing cross-user models have not yet achieved performance levels sufficient for commercialization and require users to closely adhere to a training protocol that is impractical without expert guidance. In this work, we extend a previously reported adaptive domain adversarial neural network (ADANN) to a cross-subject framework that requires very little training data from the end-user. We compare its performance to single-repetition within-user training and the previous state-of-the-art cross-subject technique, canonical correlation analysis (CCA). ADANN significantly outperformed CCA for both intact-limb (86.8–96.2%) and amputee (64.1–84.2%) populations. Moreover, the ADANN adaptation computation time was substantially lower than the time otherwise devoted to conducting a full within-subject training protocol. This study shows that cross-user models, enabled by deep-learned adaptations, may be a viable option for improved generalized pattern recognition-based myoelectric control.

## 1. Introduction

Electromyography (EMG) pattern recognition has been pursued as a way to control prosthetic devices for decades; however, there is a growing interest in its use for a wider range of commercial applications (Jiang et al., 2018). In particular, EMG is well-suited for hands-free interaction with virtual and augmented reality in consumer and industrial settings (Qian et al., 2020). This rise in interest is because EMG provides a highly intuitive control signal for motor tasks via the electrical potentials generated as muscles contract. When multiple electrodes are used, the decoded signals may be used to infer the gesture performed by the user with the help of a pattern recognition model (Oskoei and Hu, 2008). Typically, these systems require collecting EMG data from the end-user through a guided training protocol before the device is used to configure the pattern recognition model. Following this procedure, high gesture recognition accuracy can be obtained under controlled settings, as was demonstrated by Côté-Allard et al., who obtained 98.3% for a 7 class system (Côté-Allard et al., 2019). When systems are used in real-world conditions, however, performance tends to degrade substantially due to confounding factors, such as limb position, contraction intensity, and electrode shift (Scheme and Englehart, 2011; Campbell et al., 2020c; Phinyomark et al., 2020).

The most common approach to improving robustness to these confounding factors is to include these variable conditions within the training protocol. For instance, Fougner et al. (2011) demonstrated the degradation when systems are trained in a single position, but used across five different positions. By repeating the training protocol in each of those five positions, the resulting error decreased from 18 to 5.7%. Moreover, extending the training protocol to include variable conditions has been similarly validated as an solution for the effects of contraction intensity and electrode shift. When at least two contractions intensities (20 and 80% maximum contraction intensity) were supplied in the training protocol, Scheme and Englehart (2011) decreased error from 45 to 20% across a wide spectrum of intensities. Likewise, Hargrove et al. (2006) decreased error from 30 to 10% when electrodes were shifted by repeating the training protocol in five electrode locations. While these solutions enable myoelectric control to be robust to numerous sources of variability encountered in real-world conditions, extending the training protocol to all foreseeable conditions is infeasible due to the tremendous burden of time, focus, and effort for the user.

What's worse, is that this training burden is experienced each time the user puts on the device because EMG signals vary between-users (Saponas et al., 2008) and within-user across sessions and over time (Zia ur Rehman et al., 2018). EMG signals have been shown to vary with a number of factors like age (Theou et al., 2013) and physiological factors (Reaz et al., 2006) (e.g., fiber composition, blood flow, etc.). Some user groups, like amputees, have even larger inter-subject variability due to different muscle geometry and tissue filter effects (Campbell et al., 2019a). Nevertheless, these sources of variability do not completely destroy all EMG signal similarities between users (Barona López et al., 2020). This was demonstrated by Saponas et al. (2008) who achieved accuracies above chance when they pooled data from 11 able-bodied subjects to predict gestures of a different end-user. Campbell et al. (2020b) further noted that different groups of subjects were indistinguishable from one another from short windows of EMG (<300 ms) alone. Because some common information exists, cross-user models have been proposed as a method to alleviate training burden by supplementing a user's training data with added sources of variability supplied by other users.

After Saponas et al. (2008) demonstrated that pooling data from a bank of users yielded a classifier that performed better than chance for a new test user, other researchers have endeavored to adapt the pooled data to be better suited for the end-user. Matsubara and Morimoto (2013) isolated the component of feature vectors associated with gesture-variability using a bilinear transform and decreased error from 47 to 17%. Similarly, Khushaba (2014) introduced canonical correlation analysis (CCA) for gesture recognition, where each of the pooled users were first projected to the domain of an expert user to train a classifier. The mapping from an end-user to this domain was then learned using only a single repetition of each gesture. Xue et al. (2021) has further improved upon CCA by integrating optimal transport within the cross-user framework. CCA has achieved high performance for both intact-limb and amputee populations, requiring minimal data supplied by the end-user, and has improved performance when in the presence of multiple confounding factors (Cheng et al., 2018). Through these works, CCA has been established as a state-of-the-art technique for cross-user gesture recognition. Although CCA has yielded impressive results in EMG pattern recognition studies, it requires tightly controlled training protocols to ensure that an appropriate weight matrix is learned to map between end-user and expert user. This requirement limits the applicability to real-world applications, where end-users receive no researcher guidance and are unlikely to adhere perfectly to prescribed timing and progression of gestures.

A growing trend in the EMG gesture recognition literature is to employ deep learning techniques to derive features directly from data instead of using handcrafted features. Ameri et al. (2018) demonstrated that convolutional neural networks (CNN) outperformed support vector machines using existing feature sets in a functional test. Similarly, for simultaneous control of wrist motions, a CNN-based regressor again outperformed a support vector regressor in the same functional test (Ameri et al., 2019). Xia et al. (2018) introduced recurrent layers into CNNs to better leverage the time series nature of the EMG signal, resulting in a significant improvement in accuracy over the support vector regressor and standard CNN. Recurrent neural networks also inspired the recurrent and temporal fusion approaches for handcrafted feature extraction by Al Taee et al. (2020) and Khushaba et al. (2020). Similar long-short term memory networks have also provided a competitive alternative to CNN networks. He et al. (2018) found that long-short term memory networks were better suited for grasping motions, a task with strong temporal structure, than the CNN. Campbell et al. (2020a) used generative adversarial networks to simulate synthetic EMG signals to augment data sets with limited user-supplied data and better inform decision boundaries. Despite the growing body of deep learning approaches for EMG gesture recognition, very few studies have focused on cross-subject gesture recognition.

CNNs have been used for cross-subject EMG studies; however, without an adaptation strategy, cross-subject performance remains suboptimal (Park and Lee, 2016; Côté-Allard et al., 2019; Kim et al., 2020). More generally, adaptation strategies, such as transfer learning (Côté-Allard et al., 2019), supportive model selection (Kim et al., 2020), adaptive instance normalization (Li et al., 2016), and adversarial training (Ganin et al., 2016), have improved cross-user performance across a variety of applications. Although these adaptation strategies have been investigated in only a few EMG studies, they show great promise to improve cross-subject gesture recognition. For instance, Kim et al. (2020) proposed supportive model selection and demonstrated an increase in end-user accuracy over training a model with pooled training subjects by using majority vote over models trained using training subjects independently. Consequently, our objective was to develop an adaptation approach that would outperform the cross-user state-of-the-art CCA model without its inherent training restrictions.

Adaptive domain adversarial neural networks (ADANN) have recently been proposed as a powerful subject independent model (Côté-Allard et al., 2020a). In our prior work, the pooled-variance of subjects was used to create a subject-general model (one model trained and used by many subjects) that exceeded the accuracy of traditional within-subject models (one model for each subject). We have also validated it in the presence of confounding factors, such as inter-session and across day variations, using self-calibration (Côté-Allard et al., 2020b). In these works, however, each of the test users supplied a full training set, resulting in little benefit to the training burden. In contrast, the potential for ADANN to solve the cross-user problem, wherein the end-user supplies only minimal data to learn the adaptation, has not been explored.

Consequently, in this study, we explore whether ADANN can be configured as a novel cross-subject classification model that requires minimal training data from an end-user, thus alleviating training burden. The results of this work are particularly important for EMG gesture recognition in emerging consumer markets. Whereas amputee users may be sufficiently motivated to adopt myoelectric devices because of their ability to restore lost motor function, the current training burden is a substantial barrier to adoption of general consumer myoelectric devices. To improve the “out-of-the-box” experience, and facilitate widespread commercialization of EMG control systems for commercial and industrial settings, the training burden must be addressed. This work is therefore meaningful in reducing training burden, improving adoption, and growing the potential user-base of EMG gesture recognition.

## 2. Methods

The code for offline gesture recognition and statistical analyses are available at github.com/ECEEvanCampbell/TBMUDCUMFMC.

### 2.1. EMG Data

Intact-limb and amputee datasets, previously collected at the University of New Brunswick and the Shirley Ryan Lab, were adopted for this study (Hargrove et al., 2008; Scheme et al., 2011). Readers are encouraged to consult the associated manuscripts for the supplementary details of each dataset. The intact-limb dataset contained 10 subjects, and the amputee dataset contained five subjects. All subjects followed a screen-guided training protocol consisting of 16 trials of 10 gestures, where a trial consisted of a repetition of each gesture. Subjects provided the onset of motion and maintained the gesture throughout the recording period; therefore, gestures contained both the ramp-up and steady state phases of motion. As suggested by Xiang et al. (2009), data from only seven gesture classes were adopted (no movement, wrist flexion, wrist extension, wrist pronation, wrist supination, power grip, and hand open) to provide a more realistic use case for the amputee population, as amputee subjects often have difficulty producing distinct muscle patterns for classes requiring dexterous finger control (e.g., chuck grip, key grip, or pinch grip). Moreover, this gesture set may represent a more clinically-realistic or commercially-viable configuration, as it is more similar to gesture sets adopted with current devices (e.g., Myo Armband, COAPT).

Both datasets were collected using the same hardware. Ten duotrode electrodes (Myotronics, Inc.) were recorded each at 1,000 Hz using a 16-bit analog-to-digital converter. Signals were preprocessed using an analog 5th-order anti-aliasing filter with cut-off frequency of 500 Hz. For intact-limb subjects, electrodes were placed around the circumference of the forearm at the widest area with positions being as similar as possible across subjects. A similar approach was taken with amputee subjects, although electrode positions deviated slightly to best cover the residual muscle. Data were further processed using digital filters to minimize powerline interference (60 Hz notch filter) and motion artifacts (20 Hz high-pass filter). Finally, overlapping windows were extracted with window size and window increment of 151 and 50 ms, respectively.

### 2.2. Conventional Classification Using CCA

CCA is a statistical method appropriate for domain adaptation because its objective is to maximize correlation between two paired datasets: ${\left\{\left(x_{i},t_{i}\right)\right\}}_{i=1}^{n}$, where *x*_*i*_ is the *i*th *d*-dimensional sample of the expert subject dataset, and *t*_*i*_ is the *i*th *k*-dimensional sample of the target subject dataset. Within our study, *k* and *d* are the same since CCA always was performed between pairs of subjects using the same feature set. Features within the datasets are standardized to zero-mean and unit-variance to ensure that the dataset is centered and avoid the need to learn a bias term. The datasets are considered paired by organizing the datasets to have an identical progression of class labels. In modern implementations of CCA (Sun et al., 2010), a least squares regression is applied to learn an optimal linear projection *W* ∈ ℝ^*d* × *k*^ that minimizes the cost function given in Equation (1).

(1) $$\begin{array}{r} \underset{W}{\min}f\left(W\right)=\overset{n}{\underset{i=1}{\sum }}||W^{T}t_{i}-x_{i}|{|}_{2}^{2} \end{array}$$

Regularization is introduced in Equation (2) to penalize large parameter values of *W* through an additional term λ*w*_*j*_, where λ is a small positive scalar (0.04 in this study) and *w*_*j*_ is the *j*th eigenvector of *W*.

(2) $$\begin{array}{r} L_{2}\left(W,\lambda \right)=\overset{k}{\underset{j=1}{\sum }}\left(\overset{n}{\underset{i=1}{\sum }}{\left(w_{j}^{T}t_{i}-x_{i}\right)}^{2}+\lambda ||w_{j}|{|}_{2}^{2}\right) \end{array}$$

After the linear projection has converged to a solution, the data from a test user can be mapped to the space of the expert subject using Equation (3).

(3) $$\begin{array}{r} t_{x,i}=W^{T}t_{i} \end{array}$$

Following the convention proposed by Khushaba (2014), CCA was applied to learn the projections between each subject in a training set and a single expert subject, so as to minimize inter-subject variability. Once mapped, the aggregate multi-subject training dataset can be used to train a gesture classification model. A new target subject (the end-user of the device) is then accommodated by learning the projection from a single repetition of each gesture to the expert subject's domain. This projection is then applied to the remaining repetitions from the target subject prior to classifying them using the global gesture classification model. In this work, the CCA analyses were repeated for all combinations of target subjects and expert users as there is currently no accepted method of selecting the best expert user a priori. The accuracy for a target user was, therefore, reported as the mean accuracy across the different expert users.

Five handcrafted feature sets were chosen to undergo CCA projection in this analysis due to their prevalence across the literature:

- The Hudgins' time domain (TD) features are perhaps the most commonly used EMG feature set and include the mean absolute value, zero crossings, slope sign change, and waveform length features (Hudgins et al., 1993).
- The TDAR feature set provides an extension to the TD set by adding the 4th-order autoregressive coefficients.
- The TDPSD feature set was developed to increase robustness to limb position and contraction intensity by adding a combination of statistical moments and non-linear scaling; the 0th, 2nd, and 4th order statistical moments, sparseness, irregularity factor, and waveform length ratio (Khushaba et al., 2014). This feature set was originally proposed to improve robustness to limb position and contraction intensity confounding factors; however, TDPSD has since been shown to also outperform the TD feature set on controlled gesture recognition studies (Campbell et al., 2020c).
- Low sampling frequency (LSF) feature sets have been proposed as a robust alternative when data are acquired at 200 Hz (as opposed to the more traditional 1,000 Hz), although subsequent studies have demonstrated strong performance at 1,000 Hz as well. The LSF4 feature set contains maximum fractal length, l-score, mean-squared ratio, and Willison's amplitude (Phinyomark et al., 2018).
- The LSF9 feature set expands upon the LSF4 set, adding zero crossings, root mean square, integral of the EMG, difference absolute standard deviation, and variance (Phinyomark et al., 2018).

A linear discriminant analysis (LDA) classifier was adopted to classify these handcrafted feature sets due to its extensive use in EMG pattern recognition literature and low computational complexity (Campbell et al., 2019b; Leone et al., 2019).

### 2.3. Convolutional Neural Networks (CNN)

CNNs have previously been used in several within-subject EMG pattern recognition systems and achieved high performance (Zia ur Rehman et al., 2018; Ameri et al., 2019; Côté-Allard et al., 2019). The kernel employed in conventional CNN architectures allows for the spatial information from the input to be leveraged. In EMG, this consists of information from across electrodes but also in the temporal information within channels (over a short duration). A conventional CNN architecture, without adaptation, was selected for both within-subject and cross-subject evaluation in this study to provide a deep learning (DL) benchmark and provide context for the adaptation strategies employed within the ADANN network.

The employed CNN architecture contained six convolutional *blocks* followed by a single linear layer. The windows of data were first arranged into 10 × 151 (channels × samples per frame) dimensional input vectors. Within each convolutional block, the inputs were transformed by a convolutional layer that consisted of 64 kernels of size 1 × 21 (single channel × 21 samples). This produced 64 feature maps of activation values. Afterwards, these activations were regularized by batch normalization to stabilize and accelerate convergence during model training. The batch normalization transform is given in Equation (4), where *x*^(*k*)^ denotes the *k*th example within a batch, ${\hat{x}}^{\left(k\right)}$ denotes the element after regularization, μ_B_ and σ_B_ denote the mean and standard deviation of the batch, and *m* denotes the momentum term.

(4) $$\begin{array}{r} {\hat{x}}^{\left(k\right)}=\left(1-m\right)\times \frac{x^{\left(k\right)}-\mu_{B}}{\sqrt{\sigma^{2}+\epsilon }}+m\times x^{\left(k\right)} \end{array}$$

The momentum term was set to 0.99. A leaky rectified linear unit, with negative slope of 0.1, provided a non-linear mapping to segment the different blocks during training. Prior to making outputs available to the next block, dropout was implemented with a probability of 0.35 to improve generalizability of the model to unseen samples. After the six convolutional blocks, the linear layer anticipated an input of 64 × 10 (number of features × number of channels). This was transformed to a 7 × 1 output vector which represented the number of classes in the dataset. A softmax was applied to the output and the highest activated neuron was assigned as the predicted gesture.

The model hyper-parameters were selected according to values found suitable in past experiments and through iterative optimization. The learning rate was 0.04047 and the Adam optimizer was employed (Kingma and Ba, 2014). The cross entropy between the predicted and true labels was used as the loss function during training. The model was trained using a batch size of 256 windows. The learning rate decreased as training progressed according to a heuristic that monitored training and validation loss. If the loss did not achieve a new minimum within 15 epochs, the learning rate was decreased by a factor of 5, and the training procedure was considered complete when the learning rate dropped below 1e-8.

CNNs were used to test two scenarios: the difference between handcrafted and DL features within-subject and the effectiveness of DL approaches when applied across-users without domain adaptation. Consequently, CNNs were applied in both within-subject and cross-subject frameworks, where the within-subject framework was trained for each subject in isolation, and the cross-subject framework was trained on a collection of subjects and then naively applied to a new, previously unseen subject.

### 2.4. Adaptive Domain Adversarial Network (ADANN)

The ADANN followed an identical topology and training strategy to the CNN network described in the previous section, with the addition of two domain adaptation techniques: adaptive batch normalization and domain adversarial training.

*Adaptive batch normalization* is a domain adaptation technique that encodes domain-specific information in sets of batch normalization parameters and class-specific information in the weights and biases of the network (Li et al., 2016). This is achieved by associating a set of batch normalization parameters to each subject during training but using common weights and biases across all subjects. During training, the mean and variance of activations within a feature map were tracked; this resulted in 128 parameters per block being trained to encode domain information. In contrast to regular batch normalization, which is used for regularization during training but not after, the adaptive batch normalization parameters associated with a subject are retained to adapt activations after training. This enables a model that is pre-trained using a large number of subjects with multiple repetitions of gestures to be adapted to an unseen subject by learning their batch normalization parameters using a small amount of data (here, a single repetition of each gesture). In practice, this adaptation can be done using the Pytorch library by performing forward passes over the single repetition while the model is in train mode and all convolutional and linear layers are frozen (set the requires_grad attribute to false). This will update only the running mean and running variance parameters and leave the model weights unchanged. In addition to its part in previous ADANN studies, adaptive batch normalization alone has proven meaningful for EMG gesture recognition (Cote-Allard et al., 2017; Du et al., 2017).

*Domain adversarial training* is another technique used by ADANN that can improve generalization of the model to different domains (subjects) (Ganin et al., 2016; Côté-Allard et al., 2020a). Domain adversarial training relies on the network having two heads with which to simultaneously predict the elicited gesture and the subject who elicited that gesture during training. The heads consist of linear layers that operate in parallel to produce predictions on different characteristics of the data provided the same input from the convolutional blocks. These layers result in two loss terms, a gesture prediction loss ($L_{d}$) and a domain divergence loss ($L_{y}$). Standard backpropagation is used for the gesture prediction loss; however, the divergence loss is reversed (multiplied by −λ) for all convolutional blocks. In theory, this training strategy penalizes domain-specific information by regularizing across-subjects while encoding gesture-specific information. Effectively, the system is trained to be able to differentiate between gestures while being unable to differentiate between users. An appropriate penalty was observed when λ was set to 0.1, as suggested in past works (Côté-Allard et al., 2020a).

The domain adversarial training was further optimized by using only two output neurons when computing the domain divergence loss. These neurons represent whether the input comes from a particular subject or from any other subject. This strategy, as opposed to using a neuron for each subject in the training set, enabled the domain to be distinguished with a higher degree of certainty, resulting in a more appropriate penalty term. During each epoch of training, a random subject from the training set was selected as the particular subject which ensured an approximately equal representation over the course of training. Balance between the domain labels was achieved by ensuring half the batch was from the selected subject, and the remainder was from other subjects. Inputs that originated from the selected subject were issued a subject label of 1, whereas the remaining inputs were issued a label of 0. Domain divergence was computed via cross entropy between the issued labels and the predictions of the domain head.

### 2.5. Evaluation Frameworks

The performance of the classification models were validated using three different evaluation frameworks.

**Figure 1:**
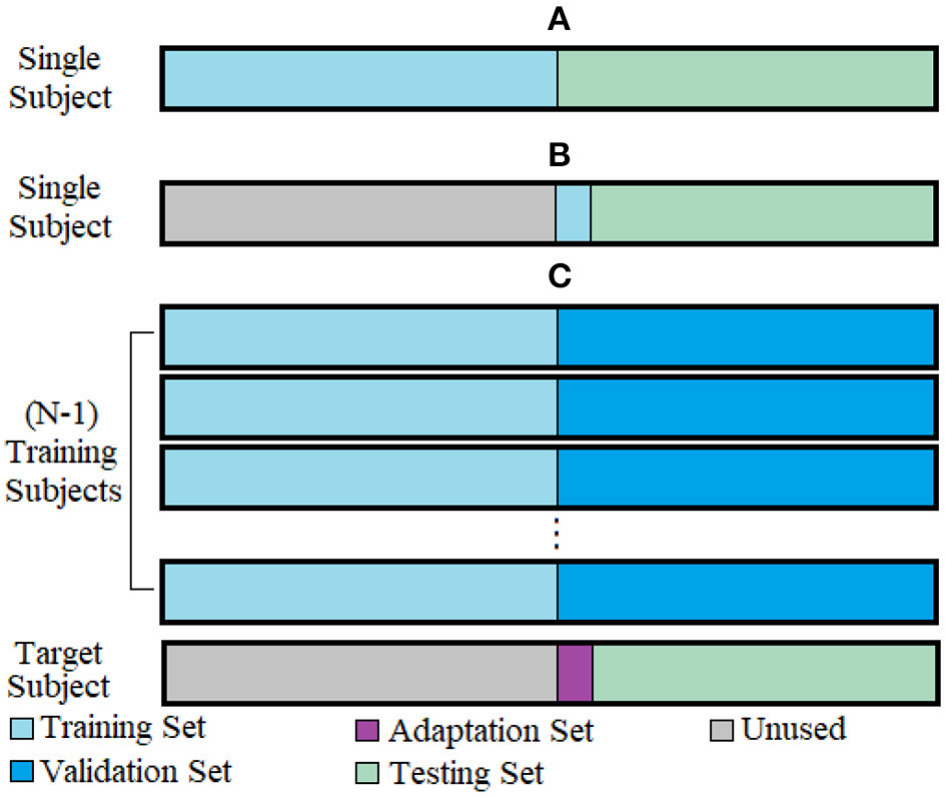
Evaluation frameworks shown for 1-fold of cross-validation. **(A)** Within-subject framework, **(B)** Single-repetition framework, **(C)** Cross-subject framework.

*Within-subject framework*: Used to establish an upper baseline for accuracy when subjects supply data using a full training protocol. The within-subject analysis was performed independently for each subject. The dataset was bisected into a training set (eight repetitions) and a testing set (eight repetitions) for each subject (Figure 1A). For this analysis, as no adaptation was necessary, the handcrafted feature sets were used without CCA, and a conventional CNN network was tested to evaluate the performance of a DL approach. The results were computed for the two groups (intact-limb and amputee populations).

*Single repetition framework*: Used to establish a lower baseline for accuracy when subjects supply only a single training repetition. The single repetition analysis was, likewise, performed independently for each subject. In contrast to the within-subject framework, only one repetition from each class was used for the training set to establish the impact of reduced training data (Figure 1B). Again, no adaptation was used for this framework, and CCA was not needed. Because of the limited amount of data, and because there were no additional repetitions available to serve as a validation set for DL, only the handcrafted feature sets were evaluated in the single repetition framework.

*Cross-subject framework*: Used to establish the benefit of using adaptation learned from a single training repetition. The amputee and able-bodied datasets were evaluated similarly, but separately, each using a leave-one-subject-out cross-validation scheme (Figure 1C). For each iteration/fold, a different subject was selected as the novel target user with the reduced training protocol. All other subjects were considered training subjects and had their data bisected into an equal training set and validation set (eight repetitions per set). In each of the ADANN, CNN, and CCA frameworks, the training set was used to establish an initial classification model for the target subject using an equal amount of data. The validation set was used in the ADANN and CNN cases to ensure that the models were not overfit by monitoring the divergence between their training and validation losses. In each fold, the target subject's data were divided into three segments: an unused segment that corresponded to the training data in other folds of the cross-validation (eight repetitions), an adaptation set that was used to learn the parameters required to adapt the model to the target subject (one repetition), and a testing set with which the accuracy of the adapted classifier was computed (seven repetitions).

### 2.6. Statistical Analyses

Statistical significance was evaluated using a two-way repeated-measures analysis of variance (RMANOVA) test with a significance value of *p* = 0.05. The 15 subjects (10 intact-limb, five amputee) were used as the repetitions within the analysis. The two independent variables under investigation were the population type (amputee, intact-limb) and the gesture recognition pipeline (23 pipelines total). If a significant difference was found at the whole study level, subsequent one-way RMANOVA tests would be conducted on these significant variables. Finally, if the one-way RMANOVA tests were significant, *post-hoc t*-tests would be conducted using a Bonferroni correction.

## 3. Results

**Figure 2:**
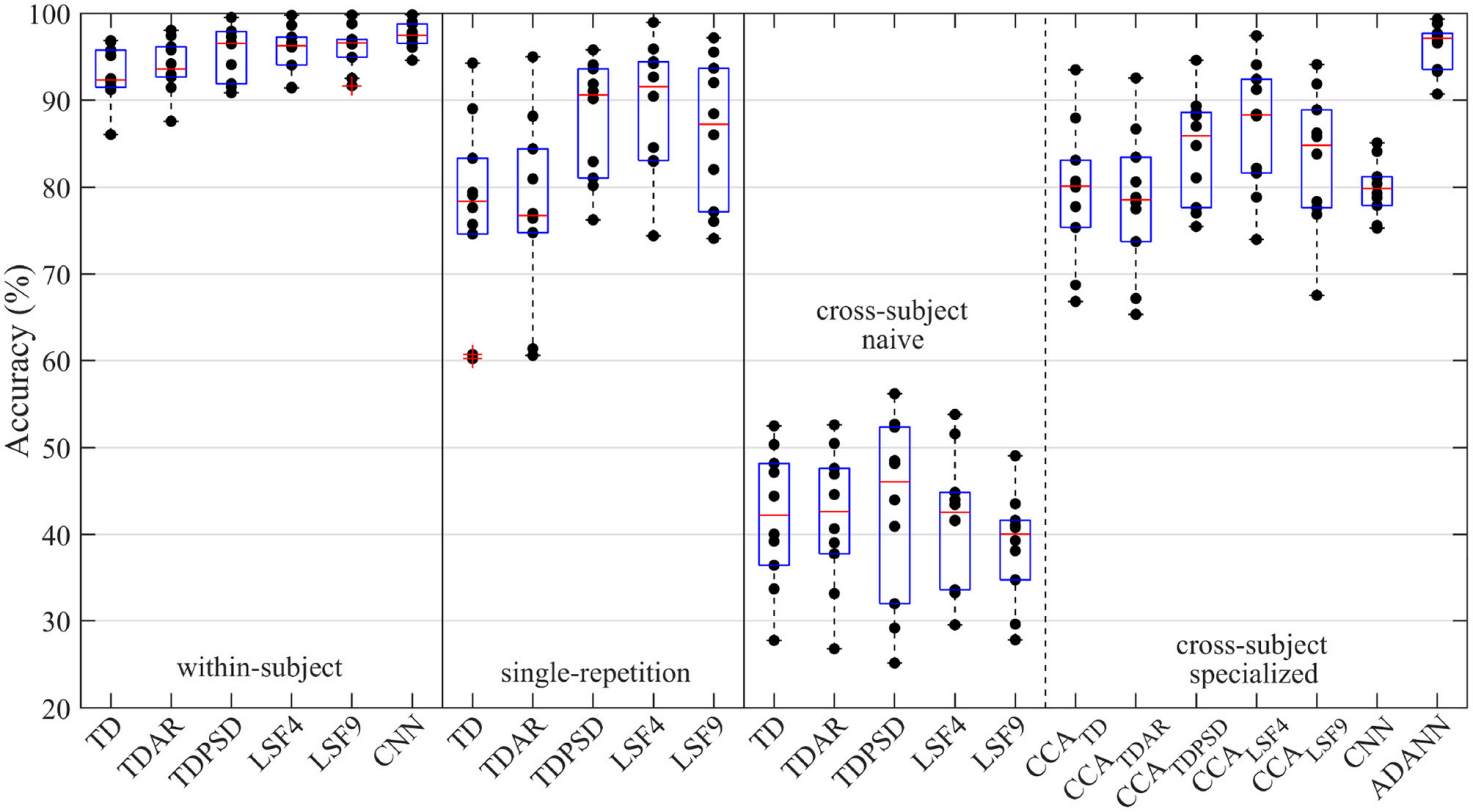
Accuracy of the intact-limb gesture recognition pipelines explored throughout the study. The pipelines were categorized according to their evaluation framework: within-subject, single-repetition, or cross-subject. The cross-subject framework was further divided into naive cross-subject pipelines and specialized cross-subject pipelines. These signified the absence or presence of a cross-subject specific solution leveraged to minimize the accuracy degradation due to between-subject variance, respectively. A dot represents the average accuracy across all gestures for a intact-limb subject using a particular gesture recognition pipeline; whereas the boxplot represents the distribution of accuracies among intact-limb subjects.

**Figure 3:**
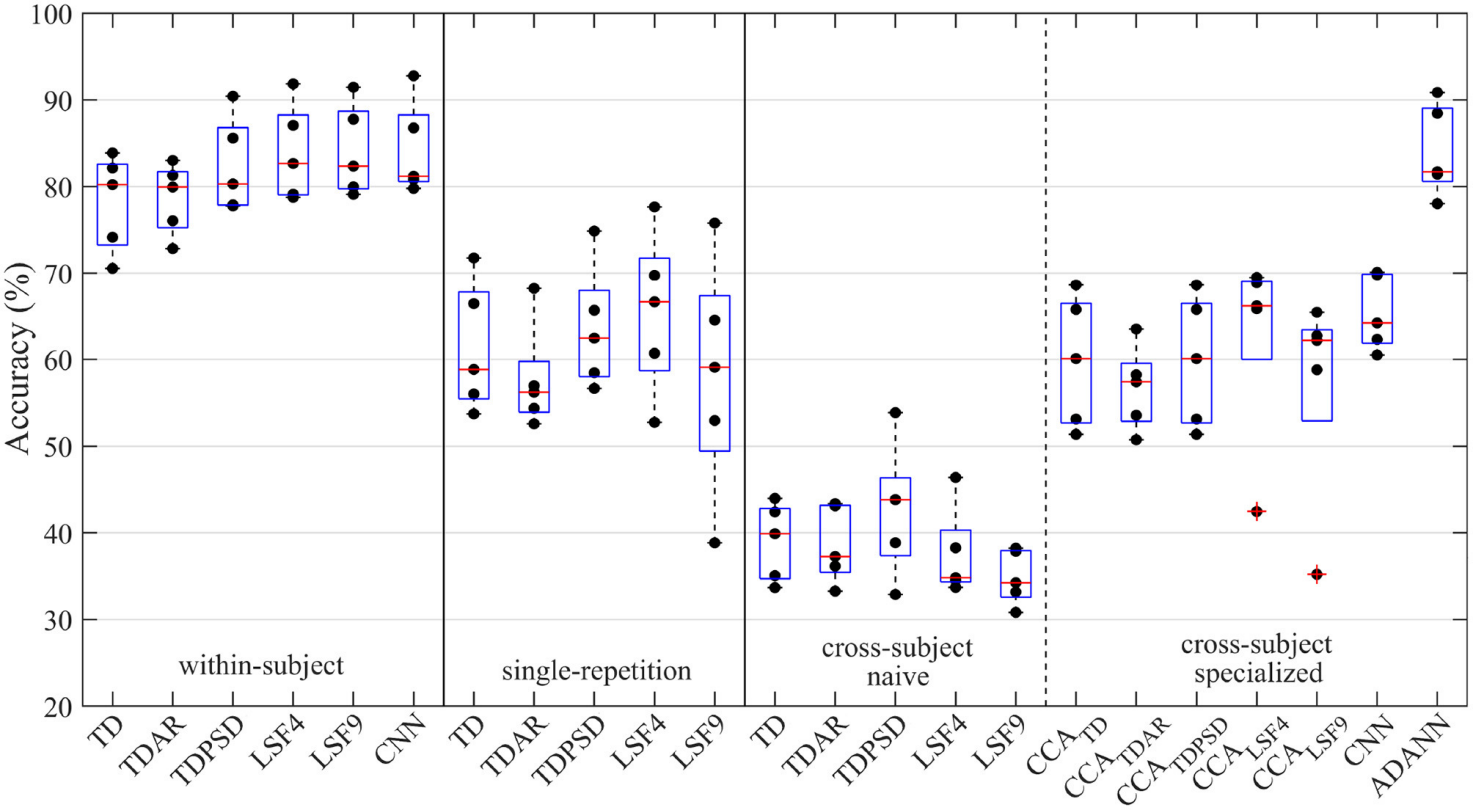
Accuracy of the amputee gesture recognition pipelines explored throughout the study. The pipelines were categorized according to their evaluation framework: within-subject, single-repetition, or cross-subject. The cross-subject framework was further divided into naive cross-subject pipelines and specialized cross-subject pipelines. These signified the absence or presence of a cross-subject specific solution leveraged to minimize the accuracy degradation due to between-subject variance, respectively. A dot represents the average accuracy across all gestures for an amputee subject using a particular gesture recognition pipeline; whereas the boxplot represents the distribution of accuracies among amputee subjects.

The performance of the within-subject, single-repetition, and cross-subject models for the intact-limb and amputee datasets are given in Figures 2, and 3, respectively.

Prior to conducting the RMANOVA, the assumptions of normality and sphericity were verified to ensure this parametric test was appropriate. The two-way RMANOVA analysis revealed a significant effect of population (*p* = 0.007), gesture recognition pipeline (*p*~0), and their interaction (*p*~0) on gesture recognition accuracy. The one-way RMANOVA investigating the effect of population found a significant effect for all pipelines except TD, TDAR, TDPSD, LSF4, and LSF9 in the cross-subject evaluation framework (*p* = 0.191, 0.181, 0.497, 0.082, 0.07, respectively).

The one-way RMANOVA investigating the effect of pipeline was found to be significant for intact-limb subjects (*p*~0). *Post-hoc* tests revealed ADANN performed significantly better for intact-limb subjects than all other pipelines across evaluation frameworks (*p* < 0.05), except the within-subject framework TDAR, TDPSD, LSF4, LSF9, and CNN pipelines (*p* = 0.058, 0.537, 0.704, 0.952, 0.174, respectively). For reference, the accuracies of the intact-limb subjects using the ADANN pipeline and within-subject TDAR, TDPSD, LSF4, LSF9, and CNN pipelines were 96.2 ± 2.8, 93.9 ± 3.1, 95.4 ± 3.1, 95.8 ± 2.7, 96.1 ± 2.5, and 97.4 ± 1.5, respectively. This indicated that despite being evaluated under the cross-subject condition, ADANN achieved an accuracy competitive with pipelines trained with full training protocols. Moreover, the ADANN pipeline resulted in consistently higher accuracies for each gesture when compared against all other intact-limb single-repetition and cross-user pipelines.

The prior state of the art cross-subject technique, CCA, also achieved reasonable cross-subject performance for intact-limb subjects. In particular, CCA_LSF4_ significantly outperformed all intact-limb cross-subject pipelines except ADANN. Interestingly, however, the accuracy obtained by CCA_LSF4_ was significantly lower than the single-repetition LSF4 pipeline (86.8 ± 7.4 vs. 89.2 ± 7.6; *p* = 0.003). This indicated that the single-repetition used for adaptation in the cross-user CCA frameworks would be leveraged better by independently training a model for this dataset.

The one-way RMANOVA investigating the effect of pipeline was also found to be significant for amputee subjects (*p*~0). The ADANN pipeline was likewise found to significantly outperform most pipelines, with the exception of the within-subject framework TDPSD, LSF4, LSF9, and CNN (*p* = 0.073, 0.804, 0.950, and 0.816, respectively). For reference, the accuracies of amputee subjects using the ADANN pipeline and within-subject TDPSD, LSF4, LSF9, and CNN pipelines were 84.1 ± 5.3, 82.4 ± 5.5, 83.9 ± 5.6, 84.1 ± 5.3, and 84.3 ± 5.5, respectively. Similar to the intact-limb results, the ADANN pipeline accuracy for amputee subjects was consistently higher for each gesture when compared against the other single-repetition and cross-user pipelines.

Amputee subjects likewise had higher accuracies while using the CCA pipelines than the untransformed naive cross-subject pipelines. In contrast to the intact-limb findings, the CCA pipelines using amputee subjects achieved statistically similar accuracies to the pipelines of the single-repetition framework.

### 3.1. Computation Time

All computations were performed in Python or MATLAB on a computer using the Windows 10 operating system with Intel i7 4790s CPU, Nvidia GTX 970 GPU, and 8 Gb RAM.

The computation times of the CCA model linear projections were 15.2, 21.1, 10.5, 14.9, and 37.6 s for intact-limb subjects and 11.8, 18.0, 8.3, 12.9, and 35.3 s for amputee subjects using the TD, TDAR, TDPSD, LSF4, and LSF9 feature sets, respectively. The computation time of the CCA adaptation was therefore suitable for myoelectric control, as this computation occurs only once per session.

The computation times for training the between-subject CNN network are 76 and 51 min for intact-limb and amputee subjects, respectively. This corresponds to the validation loss being minimized using 133 and 180 epochs for intact-limb and amputee subjects, respectively. This training procedure included no adaptation procedure, and therefore the CNN model could be immediately used by the target user with no data acquisition.

The computation times for the pre-training procedure of the ADANN network were 65 and 45 min for intact-limb and amputee subjects, respectively. This corresponds to the ADANN validation loss of these models achieving a minima after 44 and 60 epochs for intact-limb and amputee subjects, respectively. The computation time of the ADANN adaptation procedure to the end-user, however, was only 47 s. As the pre-training procedure can be performed in advance, the adaptation time is the only delay an end-user would experience from the training pipeline. This result indicates that the ADANN adaptation is completed in less time, and with far less user effort, than would be necessary to collect sufficient data for a conventional full within-subject model. The computation time of ADANN is, therefore, suitable for real-time myoelectric applications.

## 4. Discussion

Although ADANN has previously been proposed as a possible solution for subject-independent/subject-general pattern recognition (Côté-Allard et al., 2020a), this is the first successful demonstration of ADANN within a cross-user framework that substantially reduces the training burden on an end-user. The ADANN model significantly outperformed the CNN and CCA models, demonstrating the benefit of adversarial training and adaptive batch normalization when minimal training data are available. Despite the similar structure to the CNN, the use of subject-specific penalties implemented by adversarial domain training resulted in the weights of the ADANN model being more appropriate for translation to the target subject. Without this explicit penalty for subject-specific information, the CNN architecture performed similarly to the CCA models using modern feature sets. The adaptive batch normalization of the ADANN network tailored the network to the target subject while minimizing the likelihood of overfitting or underfitting given the limited data. By computing these batch normalization parameters of the target subject, the ADANN network was able to adapt under a condition that quickly achieved stability with only a single repetition of each gesture. In contrast to the pretraining procedure of the ADANN network, overfitting/underfitting of the CNN network could not be mitigated if neural weights were allowed to be modified during adaptation, as a validation set was unavailable for this process. As a result of these differences, ADANN was significantly better than the previous state-of-the-art for both intact-limb and amputee populations.

Although DL models are a growing trend in recent EMG literature (Côté-Allard et al., 2019), the conventional within-subject analysis indicated that DL did not yield a significant improvement over modern handcrafted feature sets (TDAR, TDPSD, LSF4, LSF9). Within this study, gestures were conducted in a controlled environment where limb position, contraction intensity, continuous transitions between classes, and other factors that degrade model accuracy were minimized. In studies that address these factors using DL, the flexibility of a data-driven feature extraction combined with a regularization term across conditions of the factor typically does lead to significant improvements over handcrafted models. For instance, Betthauser et al. (2019) employed a temporal convolutional network in the presence of continuous class transitions and achieved significant accuracy and responsiveness improvements over both handcrafted feature models and standard DL models (long short term memory, artificial neural network). In contrast, studies that address these factors using handcrafted feature models employ different strategies to achieve similar robustness, but require that the features, or their behavior, be explicitly defined. For instance, Amsuss et al. (2014) improved robustness to continuous transitions using a post-processing algorithm on the posterior probability of a handcrafted feature model. In short, DL models do not have an inherent advantage over handcrafted feature models for myoelectric control, although they may model characteristics not yet learned within the community with handcrafted models. Therefore, handcrafted feature sets or DL models may similarly achieve meaningful improvements over the current state-of-the-art approaches in EMG pattern recognition when an appropriate adaptation technique is applied.

Despite ADANN being by far the best solution for intact-limb and amputee cross-subject conditions, the performance for the amputee users was 12.0% lower than that of the intact-limb users. This finding was consistent across all cross-subject models evaluated, where a 19.6, 21.7, 24.3, 26.2, 20.3, and 14.5% lower accuracy was found for the CCA_TD_, CCA_TDAR_, CCA_LSF4_, CCA_LSF9_, CCA_TDPSD_, and CNN models, respectively. Scheme and Englehart (2011) noted in previous works that this decrease in accuracy is typical when models are compared between populations. A number of additional factors beyond the difference in population, however, may contribute toward this margin. First, the electrode placements were replicated less consistently across amputee participants to leverage residual muscle activity. To validate this concern, future work should investigate the performance of cross-user models when electrode placements are as similar as possible across the population or explicitly leverage the residual muscle of the amputee subjects. Khushaba (2014) proposed that CCA would be robust to changes in electrode positions between users, and provided supporting results; however, the presence of non-linear characteristics may warrant further investigation in future works. Second, the difference in the number of subjects between the populations may have magnified this effect, as the cross-subject models relied on leveraging inter-subject variability from a pool of users. To evaluate this potential, a supplementary test was conducted where the intact-limb ADANN model was limited to using the first five subjects for the initial training procedure. The resulting accuracy when less inter-subject variability was provided from these five subjects was 92.1% compared to 96.2% when 10 subjects were used. The difference in accuracy between the populations was therefore likely due to a combination of the difference in number of available subjects, the increased variability of electrode positions for amputee subjects, and intrinsic factors leading to the larger inter-subject variability between amputee subjects.

The CCA results obtained in this study do not provide a satisfactory solution to cross-user gesture recognition, despite the original CCA work finding otherwise (Khushaba, 2014). In that work, results suggested that a CCA-based cross-subject approach could outperform within-subject models trained using single repetitions. First, the between-subject CCA models all performed worse than their corresponding within-subject non-adapted models. Second, and more importantly, the between-subject CCA models did not result in a noticeable improvement over the single repetition models for any feature set. The use of CCA adaptation, instead of training a classifier with the single repetition, resulted in a reduction in accuracy of 3.2, 4.2, 5.4, 3.7, and 5.7% (1.6, 1.0, 3.8, 2.9, and 1.4%) for intact-limb (amputee) TD, TDAR, TDPSD, LSF4, and LSF9 feature sets, respectively.

In light of the large difference between the anticipated and actual effectiveness of the CCA adaptation, a follow-up analysis was conducted under similar circumstances to those used by Khushaba (2014). In Khushaba (2014), the CCA adaptation was applied on the TDPSD feature set for an amputee target user, using the other amputee subjects to aggregate the training set, however, an *intact-limb* subject was used as the expert subject. When a similar analysis was replicated here, using intact-limb expert users instead of amputee expert users, an increase in accuracy from 59.8 to 66.3% was observed. This pipeline, as prescribed by Khushaba et al., significantly outperformed all single-repetition pipelines except LSF4; further, this pipeline outperformed all cross-subject pipelines with the exception of CNN and ADANN.

This improvement was somewhat unexpected given the disparity between intact-limb and amputee populations determined in past works (Campbell et al., 2019a, 2020b). However, in Campbell et al. (2020b), subject groupings (intact-limb or amputee) could not be classified using a single window of data. Because CCA also uses a single frame at a time when computing its least squares regression, the intact-limb and amputee experts could be interchangeable in this regard. Additionally, because the CCA adaptation was computed by maximizing the correlation of the features to that of the expert user, the migratory component of the TDPSD feature set was likely minimized and, therefore, had little effect on the classifier boundaries. The improvement found when using an intact-limb expert user likely stemmed from learning a mapping from the less separable feature space of the amputee subjects to the more separable feature space of an intact-limb subject. Although class labels were not explicitly passed to the CCA adaptation, the datasets within the adaptation were always paired to represent an identical progression of gestures. The remaining disparity in model effectiveness was likely associated with differences in the classification task. In Khushaba (2014), individual finger movements and combinations of finger movements were used as the gesture set. These finer gestures are largely produced by intrinsic hand muscles, and therefore typically yield a less separable feature space, especially for amputee subjects (Xiang et al., 2009). Consequently, the projection to the more highly separable feature space of an intact-limb expert user may have allowed the CCA accuracy to exceed the within-subject accuracy of the amputee subjects. Nevertheless, the ADANN cross-subject model outperformed the CCA cross-subject model for amputee subjects on a gesture set that more similarly mirrors gestures performed when using commercial prosthetic devices.

Further studies on the ADANN architecture for cross-subject gesture recognition should be conducted to validate the results presented in this work. Although it is expected for ADANN to similarly outperform CCA in an online experiment, this subsequent analysis would allow for insights into various usability parameters. Moreover, the user-acceptance of our cross-subject model could be assessed in comparison to within-subject models. In this assessment, users would experience the difference in training burden between the cross-user model (single trial) and the within-subject model (numerous trials with breaks to minimize fatigue), and presumably achieve at least similar performance. This work did not consider the confounding factors typically encountered alongside real world applications of myoelectric control (limb position, contraction intensity, electrode shift). Consequently, future works should consider the combination of reducing training burden using ADANN while also incorporating these dynamic factors.

## 5. Conclusion

**Figure 4:**
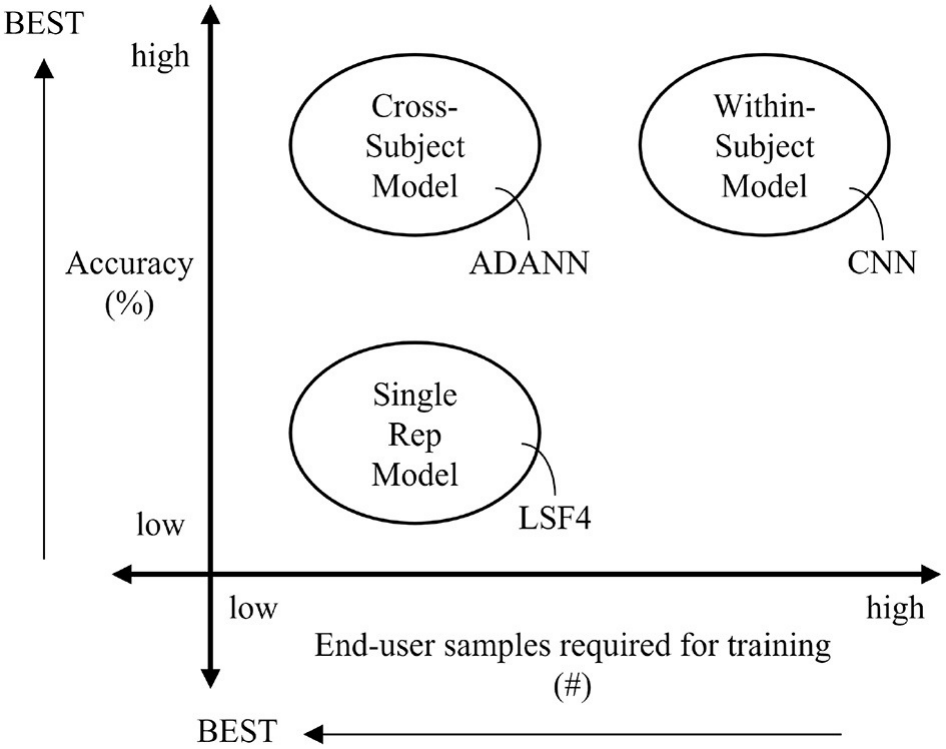
Summary of the results found in this paper, where the best performing model for each classification framework was specified. The ideal conditions for a classification model are indicated using arrows.

A visual summary of the results found in this manuscript are given in Figure 4, where the cross-subject ADANN framework was determined to be the most desirable classification model in terms of accuracy and end-user training burden. This framework resulted in significant improvements upon the prior state-of-the-art cross-subject models for both intact-limb and amputee subjects when an end-user supplied only a single repetition of each class to establish the adaptation. ADANN is, therefore, a valid approach for minimizing training burden while maintaining high accuracy. This work could enable viable rapidly-trained myoelectric control enabling widespread use across consumer and industrial applications, where training burden was previously a barrier to adoption.

## Data Availability Statement

Publicly available datasets were analyzed in this study. This data is available upon request to Erik Scheme (escheme@unb.ca).

## Ethics Statement

The studies involving human participants were reviewed and approved by University of New Brunswick Research Ethics Board. The patients/participants provided their written informed consent to participate in this study.

## Author Contributions

Access to the datasets were provided by ES. The experimental design was planned by EC, AP, and ES. The experiment and statistical analysis were carried out by EC. The manuscript was written by EC, with edits provided by AP and ES. All authors contributed to the article and approved the submitted version.

## Conflict of Interest

The authors declare that the research was conducted in the absence of any commercial or financial relationships that could be construed as a potential conflict of interest.
